# Supplementary material for: Brain mitochondrial diversity and network organization predict anxiety-like behavior in male mice
Source: Nat Commun. 2023 Aug 10;14:4726. doi: 10.1038/s41467-023-39941-0 (PMC10415311; doi:10.1038/s41467-023-39941-0)
Supplement: Supplementary file 3 — Description of Additional Supplementary Files [file 41467_2023_39941_MOESM3_ESM.pdf]

Description of Additional Supplementary Files

Supplementary Data 1. In situ hybridization RNA transcript average abundance data per brain region, from the Allen Mouse Brain Atlas accessed via The Harmonizome. Starting from all microscopic areas with in situ gene expression data (n=2,170), areas that correspond to each of our target brain areas were selected, which are listed on the tab labeled 'Included_subregions_list'. These subregions were then averaged to obtain a single expression value for each gene, per brain area, which is listed on the tab labeled 'Regional_average_gene_expression'.

Supplementary Data 2. Differential gene expression analysis between the three identified networks. Expression values for each gene per brain area were averaged across brain regions for each of the networks (n=6 areas), network 2 (6 areas), and network 3 (4 areas) to create average gene expression values for each network. Genes that are labeled as upregulated are overexpress by >100% (double) using a threshold of one Log2 unit, and genes that are labeled as downregulated are underexpressed by >50% (half) in that network relative to the other two networks.

Supplementary Data 3. A markdown file containing code and figures shown in Figure 4. This file also contains additional information (tables, interactive plots) supporting the analysis. This data utilized the Allen mouse brain atlas dataset and human MitoCarta3.0.
